# Comparison of interstitial high-dose-rate brachytherapy and stereotactic radiotherapy in breath-hold technique for inoperable primary and secondary liver tumors

**DOI:** 10.1016/j.phro.2025.100811

**Published:** 2025-07-13

**Authors:** Philipp Geissler, Saskia Spautz, Kathrin Hering, Isabell Seiler, Frank Heinicke, Ilias Sachpazidis, Dimos Baltas, Sebastian Schäfer, Christos Moustakis, Nils H. Nicolay, Franziska Nägler

**Affiliations:** a Department of Radiotherapy, University Hospital Leipzig, Leipzig, Germany; b Comprehensive Cancer Center Central Germany, Partner Site Leipzig, Leipzig, Germany; c Division of Medical Physics, Department of Radiation Oncology, Medical Centre - University of Freiburg, Medical Faculty - University of Freiburg, German Cancer Consortium (DKTK), Partner Site DKTK, Freiburg, Germany

**Keywords:** Hepatocellular carcinoma, Liver metastases, Radiotherapy, Stereotactic radiotherapy, Brachytherapy, Plan comparison

## Abstract

**Background and purpose:**

In recent years, ablative radiotherapy of primary and secondary liver tumors has become a highly regarded treatment option. We aimed to compare high-dose-rate interstitial brachytherapy (HDR-iBT) and stereotactic body radiotherapy (SBRT) in free breathing (FB) and deep inspiration breath hold (DIBH) in order to support future decisions on the appropriate radiation technique for liver tumors.

**Materials and methods:**

Forty-two patients with 1–4 liver malignancies undergoing HDR-iBT with 1x15-20 Gy were included. Eighty-eight SBRT plans in DIBH or FB with a prescription dose of 3x12.5 Gy to the surrounding 67 % isodose were generated using the HDR-iBT-computed tomography. Dose-volume parameters according to ICRU91 regarding organs-at-risk (OAR), target coverage, generalized equivalent uniform dose (gEUD_2Gy_), biological dose parameters, dose uniformity, conformity and plan quality indices were compared.

**Results:**

The mean target doses for HDR-iBT were at least twice as high as in SBRT, but target coverage was significantly more conformal for SBRT and the gEUD_2Gy_ was higher (*p* < 0.001). The healthy tissue conformity indices (HTCI) for SBRT_FB_ and SBRT_DIBH_ were significantly higher than for HDR-iBT (*p* < 0.001). For all techniques, a significant positive correlation between HTCI and target volume size was found. HDR-iBT achieved lower mean and maximum doses for most investigated OAR-parameters. Comparing SBRT_DIBH_ and SBRT_FB_, all OAR-parameters presented significantly lower values for SBRT_DIBH_ (*p* < 0.001).

**Conclusion:**

While SBRT_DIBH_ achieved a more conformal target coverage with both higher minimal doses and gEUD_2Gy_, brachytherapy offered higher mean and maximal doses to the target volume and a better sparing of OAR, depending on optimal catheter positioning.

## Highlights

• Average dose to the liver tumor was increased by a factor two with brachytherapy.
• Brachytherapy achieved optimal sparing of gastrointestinal organs.
• Stereotactic radiotherapy offered higher target coverage and conformity.
• Breathhold reduced organ at risk doses compared to free breathing.

## 1 Introduction

With increasing numbers of malignant liver tumors, hepatocellular carcinoma (HCC) remains the most common liver malignancy globally [1]. The global incidence of liver malignancies in 2022 was approximately 860000, with a global mortality of about 750,000 annually [1].

Liver resection and transplantation are potentially curative treatment options, whereby liver transplantation demonstrated the best overall survival rates for patients with tumors confined to the liver [2,3]. However, advances in surgical and local ablative procedures and systemic therapies led to improvements in local control and, in some cases, increasing survival rates. Various local therapies can be used as bridging approaches before liver transplantation, with studies showing delayed tumor progression, lower deregistration rates from the transplant waiting list and better survival rates after liver transplantation [4].

Numerous local treatments are also available for inoperable HCC, like radiofrequency ablation (RFA), microwave ablation (MWA) or transarterial chemoembolization (TACE) that has shown a moderate improvement in survival compared to best supportive care in a recently published study [5]. For some patients, these approaches are not suitable due to tumor size or localization. Encouraging results have also been demonstrated for stereotactic body radiotherapy (SBRT) in the treatment of inoperable or recurrent HCC, with excellent local control rates and low toxicity, for example compared to Sorafenib or TACE [[6], [7], [8], [9]].

In the context of oligometastatic disease (OMD), SBRT is increasingly used for unresectable liver metastases, with reasonable local control rates depending on the applied dose [[10], [11], [12]]. In a recently published *meta*-analysis including HCC and liver metastases, two-year local control was significantly higher for SBRT of liver metastases compared to RFA (84 % vs. 60.0 %), but there was no significant difference for treatment of HCC [13]. Very promising local control rates were also reported for high dose-rate interstitial brachytherapy (HDR-iBT), not only for bridging to transplant or inoperable HCC, but also in the context of unresectable liver metastases in oligometastastic, oligopersistent or oligoprogressive disease, even for multiple pre-treated patients [[14], [15], [16], [17], [18], [19], [20]].

To our knowledge, the literature for plan comparisons of different radioablative techniques such as SBRT, HDR-iBT or kilovoltage electronic brachytherapy (eBT) is scarce and heterogeneous [[21], [22], [23], [24], [25], [26]]. Thus, we aimed to compare clinically delivered liver HDR-iBT plans with free-breathing or breath-hold SBRT plans to evaluate potential differences. Dose-volume parameters for target volumes and organs-at-risk (OAR) were analyzed to support clinical decision-making in the development of local radiotherapeutic strategies for these challenging tumors.

## 2 Materials and methods

### 2.1 Patient selection

We reviewed 133 consecutive patients with primary or secondary liver malignancies receiving single-fraction HDR-iBT at a tertiary cancer center between 2015 and 2024. Patients with MR-guided liver HDR-iBT were excluded due to the lack of planning computed tomography (CT). Inclusion criteria were one to four liver lesions suitable for both HDR-iBT and SBRT, sufficient liver function (Child-Pugh Scores A/B) and no relevant ascites (contraindication for HDR-iBT). 42 patients with clinically delivered HDR-iBT-plans were selected for re-planning with SBRT.

Patient characteristics are shown in Table 1. The majority of HDR-iBT procedures was carried out using a single dose of 20 Gy (n = 26). Median number of implanted catheters for HDR-iBT was 2 (range: 1–7). This study was reviewed and approved by the local Ethics Committee (432/24-ek).

**Table 1:** Patient characteristics.

**Variable**	**N (range)**
*Gender*	
Female	12
Male	30
*Age*	
Mean	67 (39–81)
*Primary liver malignancies*	
HCC	25 patients, 33 lesions
CC	5 patients, 5 lesions
*Child-Pugh (CP)score*	
A	24
B	5
not specified	1
*Secondary liver malignancies*	
Metastases	12 patients, 18 lesions
CRC	5
Breast Cancer	3
Melanoma	2
Others	2
*HDR-iBT plan dose*	
15 Gy	14
16 Gy	3
18 Gy	1
20 Gy	26
Median dose (Gy)	20 (range: 15–20)
*Median number of lesions*	1 (range: 1–4)
*Median target volume HDR-iBT (cm^3^)*	30.5 (range: 1.3–174.9)
*Median target volume SBRT*	
Free breathing PTV (*cm^3^*)	131.7 (range: 20.7–434.3)
Deep inspiration breath hold PTV (*cm^3^*)	75.7 (range: 8.6–281.7)
*Number of catheters for HDR-iBT, median*	2 (1–7)

### 2.2 Interstitial HDR brachytherapy (HDR-iBT)

After catheter placement in Seldinger technique (Afterloadingkatheter, Primed® Halberstadt Medizintechnik GmbH) under CT-guidance, contrast-enhanced planning CT images with a slice thickness between 3 and 5 mm were acquired according to the discretion of the interventional radiologist and transferred to the treatment planning system (TPS) Oncentra Brachy versions 4.3, 4.6 (Elekta AB, Stockholm, Sweden). Structures of interest were delineated by a board-certified radiation oncologist including gross tumor volume (GTV_iBT_) and OARs as listed in Supplementary Table S1. Since applicators had fixed positions within the tissue, no margin was added, and the macroscopic tumor (GTV_iBT_) was considered the planning target volume for brachytherapy (PTV_iBT_). A medical physicist performed catheter reconstruction and treatment planning based on the task group 43 (TG-43) algorithm [27] with a dose grid resolution of 1 mm. 15–20 Gy were prescribed to 100 % of the GTV_iBT_, and the dose was modified to comply with the OAR dose constraints, accepting dose reductions for the target volume if necessary (Supplementary Table S1) [28]. HDR-iBT was carried out with a microSelectron afterloader (Elekta AB, Stockholm, Sweden) equipped with an Iridium-192 source. The reported treatment time was evaluated excluding the time required for catheter placement and treatment planning.

### 2.3 Virtual SBRT re-planning

**Fig. 1:**
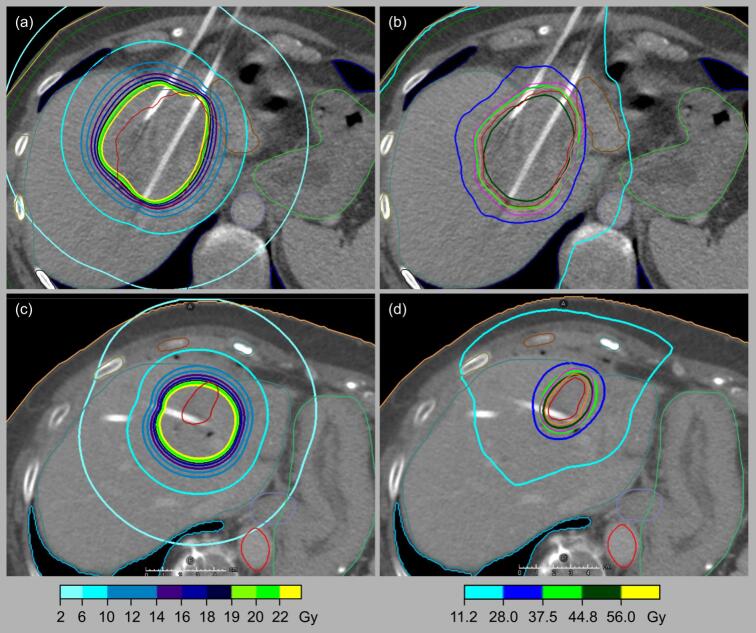
Dose distribution for a patient with one lesion and (a) clinically applied HDR-iBT, (b) virtual replanned SBRT_DIBH_ with advantages for HDR-iBT, because of its steeper gradient to OAR. Second example with (c) clinically applied HDR-iBT and advantages for (d) SBRT_DIBH_, because of better conformity.

For virtual SBRT re-planning of each lesion in DIBH and FB, initial CT scans in breath-hold were used as routinely obtained for HDR-iBT planning (Fig. 1). CT images and structure sets were imported to the Raystation planning system (version 12A; Raysearch Laboratories AB, Stockholm, Sweden). For virtual SBRT re-planning, the original GTV_iBT_ was used as GTV_SBRT_. In order to consider respiratory mobility of livertumors during SBRT_FB_, a respiration-correlated CT (4D-CT) was simulated with additional safety margins to the GTV_iBT/SBRT_. A 10 mm margin was added cranio-caudally, and 5 mm were added in all other directions to generate a virtual internal target volume (ITV_SBRT_FB_) and PTV expansion amounted to 3 mm in all directions (PTV_SBRT_FB_) [29]. For SBRT_DIBH_, a clinical target volume (CTV_SBRT_DIBH_) instead of the ITV_SBRT_FB_ was generated by adding a 2 mm margin to the GTV_iBT/SBRT._ PTV_SBRT_DIBH_ margins were identical to PTV_SBRT_FB_ margins. All SBRT plans were computed for a LINAC TrueBeam STX (Varian Medical Systems, Palo Alto, CA) with a 120-leaf MLC and 6MV flattening-filter-free photons. The Collapsed Cone algorithm (CCDose v5.4) was used for dose calculation. A dose of 3x 12.5 Gy was prescribed to the surrounding 67 % isodose, resulting in a dose maximum of 55.97 Gy, and 95 % of the PTV was covered by the 95 % isodose of the prescribed dose [11,30]. Because of the limited slice thickness of the HDR-iBT-planning CTs, a 3 mm dose grid was used. OAR dose constraints for SBRT are shown in Supplementary Table S1 [31]. The reported treatment times for SBRT were derived from the beam-on times of the TPS.

### 2.4 Comparison of dose-volume parameters of HDR-iBT and SBRT_FB_/ SBRT_DIBH_

Dose parameters for target volumes (GTV_iBT_, PTV_SBRT_FB_, PTV_SBRT_DIBH_) were reported according to ICRU91 [32,33] as D_2%_, D_50%_, D_90%_, D_98%_, D_99.9%_ and maximum dose in 0.03 cm^3^ (or 1 cm^3^ as commonly used for HDR-iBT), each in absolute and relative values of the prescribed dose. For comparison of the two different dose concepts, mean doses of the respective target volumes were calculated considering the biological effective dose (BED), the equivalent dose in 2 Gy (EQD2) and the generalized equivalent uniform dose (gEUD_2Gy_), all with *α/β* = 10 Gy for liver tumors and the volume effect *α = -10* [[34], [35], [36], [37], [38]]. The gEUD_2Gy_ was calculated voxelvise in the TPS. Indices for target conformity (CI), healthy tissue conformity (HTCI) and conformation number (CN) were calculated according to the definitions in Supplementary Table S2 [[35], [36], [37],^39^,^40^]. Doses to OAR were evaluated for each treatment technique and reported in EQD2 dose values (with *α/β* = 3 Gy) or in relative values of the prescribed dose. For the liver, multiple dose-volume parameters were evaluated based on previous studies [41].

### 2.5 Statistical analysis

All statistical evaluations were performed using IBM SPSS Statistics version 29 (IBM Deutschland GmbH, Böblingen, Germany). Statistical comparisons of dose–volume parameters were conducted using two-sided paired Wilcoxon signed-rank tests across the patient cohort, with a significance level of *α* = 0.05. The correlation of CI and HTCI with the respective target volume was investigated with a Spearman-Rho test (two-sided, significance level of *α* = 0.05). The resulting *p*-values were considered significant for *p* ≤ 0.05.

To ensure the quality of our plan comparison study, we used retrospectively the score sheet of Radiotherapy Treatment plannINg study Guidelines (RATING) and achieved a score of 86 % [42].

## 3 Results

### 3.1 Plan delivery characteristics

Both SBRT techniques had comparable simulated treatment delivery times for one fraction (median: 2.0 min, range: 1.5–4.8 min) that were, even if 3 fractions have to be considered in clinical routine, significantly shorter than those for HDR-iBT (median: 24.0 min, range: 5.0–48.0 min). The range of HDR-iBT delivery times was caused by variations of the radiation source activity. However, delivery times for SBRT techniques were estimated by the TPS as beam-on times only, not considering the time needed for patient positioning or prolonged delivery times for breath-hold procedures in case of SBRT_DIBH_.

### 3.2 Target volume coverage for HDR-iBT and SBRT approaches

**Fig. 2:**
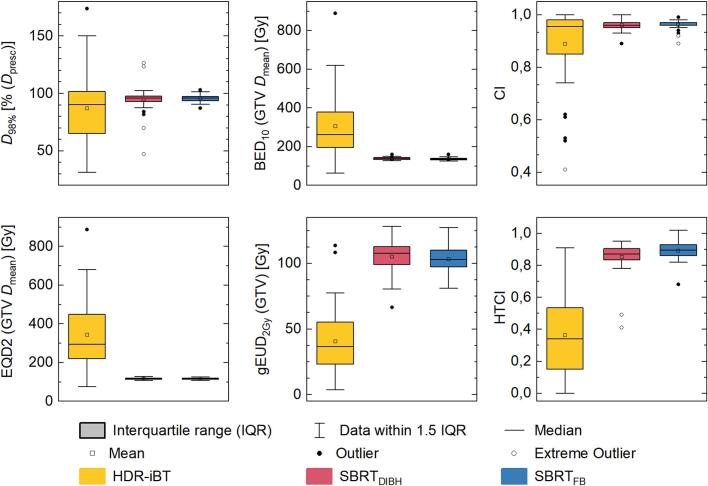
Boxplots regarding target volume dose evaluations of HDR-iBT (yellow), SBRT_DIBH_ (red) and SBRT_FB_ (blue) treatment plans: target coverage (D_98%_; upper left) relative to the prescribed dose (D_presc_), equivalent dose to 2 Gy (EQD2) of the mean GTV dose (D_mean_; lower left), biological effective dose (BED_10_) of the mean GTV dose (upper middle), generalized equivalent uniform dose (gEUD_2Gy_) in the GTV (lower middle), conformity index (CI; upper right) and healthy tissue conformity index (HTCI; lower right). (For interpretation of the references to colour in this figure legend, the reader is referred to the web version of this article.)

Target volume coverage for both SBRT concepts was more conformal than for HDR-iBT, demonstrated by a higher median D_98%_ of 96 % (range: FB: 87–103 %; DIBH: 47–127 %) compared to HDR-iBT (D_98%_ 90 %; range: 31–174 %) (Fig. 2). These differences were significant for SBRT_FB_ vs. HDR-iBT D_98%_ (*p* = 0.029) and showed a trend for SBRT_DIBH_ (*p* = 0.093). Considering D_50%_, D_2%_ and D_max_, significantly higher values were achieved with HDR-iBT than with SBRT techniques.

HDR-iBT offered significantly higher values for mean target dose calculated in EQD2 (293.1 Gy; range: 75.1–887.8 Gy) and BED_10_ (262.6 Gy; range: 62.6–889.5 Gy), compared to SBRT_FB_ (EQD2: 115.2 Gy; range: 107.0–125.1 Gy; BED_10_: 135.4 Gy; range: 124.7–160.1 Gy) and SBRT_DIBH_ (EQD2: 116.2 Gy; range: 107.0–127.2 Gy; BED_10_: 138.4 Gy; range: 127.9–159.9 Gy) (p < 0.001). Comparing gEUD_2Gy_ for HDR-iBT (36.5 Gy; range: 3.6–113.7 Gy), SBRT_FB_ (103.0 Gy; range: 81.0–127.4 Gy) and SBRT_DIBH_ (107.5 Gy; range: 66.5–128.2 Gy), higher values for both SBRT techniques were achieved (p < 0.001). Absolute and relative values of dose coverage are shown in Supplementary Table S3.

### 3.3 Conformity indices for HDR-iBT and SBRT approaches

**Fig. 3:**
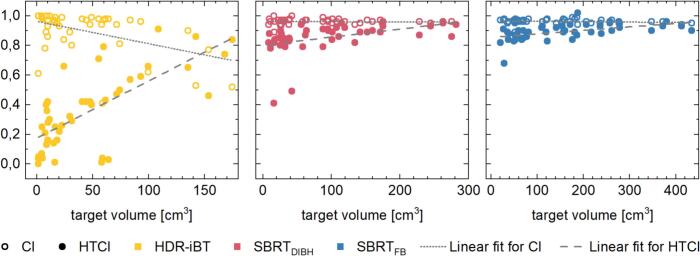
Scatter plots of the target volume size in correlation to both the conformity index (CI; empty circle) and the healthy tissue conformity index (HTCI, filled circle) for the HDR-iBT (yellow), SBRT_DIBH_ (red) and SBRT_FB_ (blue) treatment plans. Linear fits of the correlating data are indicated for both the CI (dotted line) and the HTCI (dashed line), respectively. (For interpretation of the references to colour in this figure legend, the reader is referred to the web version of this article.)

While the median CI of 0.96 was identical for all techniques, HDR-iBT plans reached significantly lower CI (range: 0.41–1.00) compared to both SBRT approaches (range: 0.89–1.00) (p = 0.016). Moreover, a negative correlation between CI and target volume size was observed for HDR-iBT (−0.47; p = 0.001), whereas no correlation was found for SBRT (Fig. 3).

HTCI for SBRT_FB_ (median: 0.90; range: 0.68–1.02) and SBRT_DIBH_ (median: 0.87; range: 0.41–0.95) was significantly higher than for HDR-iBT (median: 0.34; range: 0–0.91) (p < 0.001). For all approaches, a positive correlation between HTCI and target volume size was found, although the correlation was stronger for HDR-iBT (0.71) than for SBRT_FB_ (0.57) and SBRT_DIBH_ (0.62) (p < 0.001) (Fig. 3).

Considering the product of CI and HTCI, significantly higher CN was achieved with SBRT_FB_ (median: 0.86; range: 0.66–0.97) compared to SBRT_DIBH_ (median: 0.84; range: 0.41–0.92) and HDR-iBT (median; 0.33, range: 0.00–0.82) (p < 0.001).

### 3.4 Dose distribution in liver and uninvolved liver volume

**Fig. 4:**
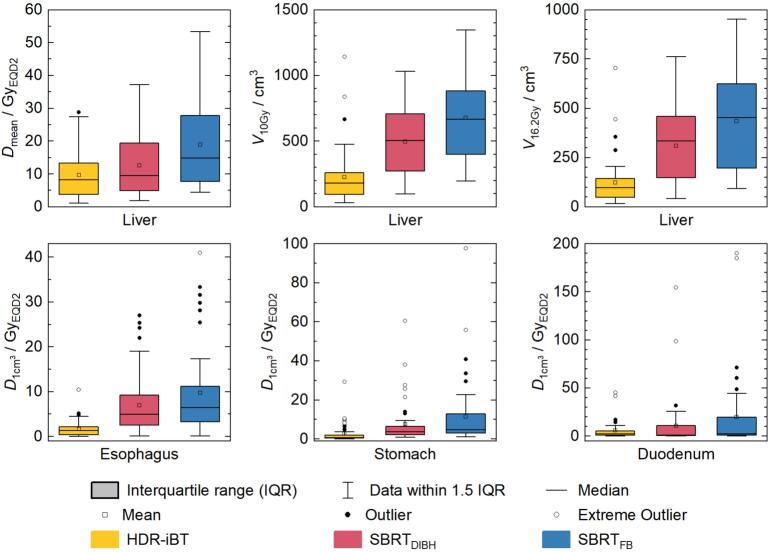
Boxplots regarding dose evaluations of selected organs at risk parameters of HDR-iBT (yellow), SBRT_DIBH_ (red) and SBRT_FB_ (blue) treatment plans: mean dose (D_mean_) in liver (upper left), liver volume exposed to 10 Gy (V_10Gy_; upper middle) and 16.2 Gy (V_16.2Gy_; upper right), maximum dose (D_1cm_^3^) in esophagus (lower left), stomach (lower middle) and duodenum (lower right). All reported dose values were calculated in equivalent dose 2 Gy (Gy_EQD2_). (For interpretation of the references to colour in this figure legend, the reader is referred to the web version of this article.)

For all evaluated liver dose-volume parameters except D_66%_<10 Gy, significantly lower values were achieved for HDR-iBT (median V_10Gy_: 11.6 %; range: 1.5–70.3 %; V_16.2Gy_: 5.9 %; range: 0.8–43.4 %) compared to SBRT_FB_ (V_10Gy_: 39.1 %; range: 12.1–83.5 %; V_16.2Gy_: 24.7 %; range: 5.9–70.7 %) and SBRT_DIBH_ (V_10Gy_: 28.5 %; range: 5.6–73.4 %; V_16.2Gy_: 17.0 %; range: 2.0–55.2 %). Although HDR-iBT achieved significantly lower D_66%_ (median: 1.4 Gy_EQD2_; range: 0.2–29.7 Gy_EQD2_) compared to SBRT_FB_ (median: 1.7 Gy_EQD2_; range: 0.2–30.8 Gy_EQD2_), no significant difference was found for the comparison with SBRT_DIBH_, reaching the lowest D_66%_ overall (median: 0.7 Gy_EQD2_; range: 0.1–20.2 Gy_EQD2_). For liver D_meanEQD2_, there were smaller but significant differences between HDR-iBT with median 8.2 Gy_EQD2_ (range: 1.1–28.8 Gy_EQD2_) and SBRT_DIBH_ with median 9.5 Gy_EQD2_ (range: 1.8–37.2 Gy_EQD2_). Comparing SBRT_DIBH_ and SBRT_FB_, all liver dose-volume parameters presented significantly lower values for SBRT_DIBH_ (Fig. 4, Supplementary Table S4).

### 3.5 Dose distribution in gastrointestinal OARs

HDR-iBT-plans achieved better results with significantly lower dose maximum in 1 cm^3^ in most gastrointestinal OARs such as esophagus, stomach, gall bladder and large intestine (Supplementary Fig. S1). SBRT_DIBH_ plans delivered lower maximum dose to the duodenum (median: 1.1 Gy_EQD2_; range: 0.0–154.6 Gy_EQD2_) compared to HDR-iBT, but reaching significance only for D_meanEQD2_ (p = 0.015). Lower maximum doses to the remaining small intestine (without duodenum; median: 0.3 Gy_EQD2_; range: 0.0–56.8 Gy_EQD2_) were observed for HDR-iBT compared to SBRT_FB_ (median: 0.8 Gy_EQD2_; range: 0.0–162.9 Gy_EQD2_) (p ≤ 0.013).

Regarding the mean dose to OAR relative to the respective dose prescription (Presc), significantly lower values were achieved by HDR-iBT for stomach (median: 2.1 %_Presc_; range: 0.6–11.5 %_Presc_) and esophagus (median: 6.1 %_Presc;_ range: 0.9–19.6 %_Presc_) compared to SBRT_FB_ (p < 0.001 for both) and SBRT_DIBH_ (p < 0.001 for stomach). However, for the duodenum, HDR-iBT plans led to significantly higher relative doses compared to SBRT_DIBH_ (p < 0.001) or SBRT_FB_ (p = 0.056) (Supplementary Table S4).

### 3.6 Dose distribution in other OARs

Lower maximum dose values in skin, descending aorta and spinal cord were observed for HDR-iBT compared to both stereotactic approaches (p ≤ 0.001). Regarding the heart, HDR-iBT achieved significantly lower maximum doses compared to SBRT_FB_ (p ≤ 0.001), but dose differences were not signficant compared to SBRT_DIBH_ (p = 0.073). Lower mean dose values were achieved in the right kidney by both SBRT_FB_ (median: 2.2 %_Presc_; range: 0.0–29.1 %_Presc_) and SBRT_DIBH_ (median: 1.1 %_Presc_; range: 0.0–26.6 %_Presc_) compared to HDR-iBT (median: 4.8 %_Presc_; range: 0.7–25.8 %_Presc_) (p ≤ 0.01) (Supplementary Table S4).

## 4 Discussion

In the last decade, focal ablative radiation therapy of primary and secondary liver tumors has developed into a highly regarded treatment option. To our knowledge, this is the first comparative study for local ablative radiotherapy of liver malignancies investigating breath-hold-optimized liver SBRT. We found significantly better sparing of OAR for SBRT_DIBH_ compared to SBRT_FB_. Additionally, SBRT offered significantly higher target volume coverage and conformity, and three times higher gEUD_2Gy_ compared to HDR-iBT. In contrast, HDR-iBT achieved twice as high average doses in EQD2 and BED_10_ and superior sparing of OAR.

However, data on plan comparisons of different ablative radiation techniques are scarce. There are two studies comparing virtually planned SBRT (1–3 fractions) with HDR-iBT for primary liver malignancies [21,22]. In addition, three other studies investigated re-planning approaches for local external radiotherapy of liver metastases [[23], [24], [25]] and one plan comparison of different radiotherapy modalities including SBRT, HDR-iBT and eBT for non-resected oligometastatic liver disease [26]. Most of them suggested advantages for target volume coverage while sparing liver volume for HDR-iBT vs. SBRT_FB_. Additionally, in our plan comparison, due to smaller planning target volumes of SBRT_DIBH_, all OAR dose-volume parameters presented significantly lower values for SBRT_DIBH_ compared to SBRT_FB_ and even demonstrated favorable OAR exposure compared to HDR-iBT for liver D_66%_ relative to prescribed dose and mean EQD2 dose for duodenum and right kidney.

As also reported previously, we observed higher doses to target volumes and better sparing of most OARs for HDR-iBT [21,22,24,25]. Dose maxima of HDR-iBT were mainly located inside or on the surface of the applicator and not within the tumor tissue itself. Therefore, we focused on the mean target doses for our evaluations. In our analysis, mean target EQD2 and BED were at least twice as high for HDR-iBT compared to free-breathing and breath-hold-optimized SBRT. Significantly higher gEUD_2Gy_ were found for SBRT. This effect was due to the greater consideration of insufficiently covered areas in the target volume in this calculation. This also underlines the importance of full coverage of the target volume in HDR-iBT. Despite the fact that there is no consensus on the required dose even in recently published guidelines for HCC by the ISRS [43,44], 3-fraction SBRT used for this analysis reached the recommended BED > 100 Gy for liver malignancies [12,[43], [44], [45], [46]]. Further clinical investigations are necessary to define the best approach regarding optimal prescribed dose.

In recent guidelines, the European Society of Medical Oncology (ESMO) considers SBRT a locally ablative treatment option for metastatic colorectal carcinoma, and both SBRT and HDR-iBT as treatment options for recurrent HCC [18,47], The American Association for the Study of Liver Diseases (AASLD) also recommends liver SBRT as a treatment option for HCC, achieving durable local control in patients unsuitable for resection [48]. Additionally, unlike thermal ablation approaches, radiation therapy is not limited by the tumor proximity to large vessels.

Regarding dose coverage in our study, D_98%_ was better in the respective SBRT plans, as also shown by Bilski et al. [25], but in our analysis also resulted in significant higher gEUD_2Gy_, CI, HTCI and CN for both SBRT_FB_ and SBRT_DIBH_. However, evaluated HTCI for HDR-iBT was highly dependent on the catheter position. In case of catheter placement at the edge or even outside the target volume, acceptable coverage could only be achieved by an overall increase of the irradiated volume, which also increases the dose exposure to nearby OARs, especially the surrounding liver. In agreement with the above-mentioned studies, we also noted a better sparing of most OARs for HDR-iBT. Better OAR protection of HDR-iBT resulted both from a steeper dose gradient and from smaller target volumes. While for SBRT, median breathing-induced motion amplitudes of 3 mm (1–14 mm) were reported despite the use of breath control approaches, HDR-iBT does not require respiratory-dependent margins [49].

Due to the retrospective study design, the current study has some limitations, especially regarding a potential selection bias, as all patients in this analysis were selected as suitable for brachytherapy. Additionally, due to the usage of available HDR-iBT planning CTs for our analysis, the dose grid was not optimized for SBRT, and only limited information about breathing motion was available. We tried to overcome the aforementioned limitation by adding safety margins recommended in literature and simulating an ITV concept for SBRT_FB_ [29]. Another possible limitation may be due to the use of TG-43 dose calculation for treatment planning for HDR-iBT, which accounts neither for tissue heterogeneity nor for full scatter conditions, and previous studies suggest that there may be an overestimation of the dose at superficial regions or regions close to large air cavities [50,51].

In conclusion, HDR-iBT is a valuable method for local ablative treatment of liver lesions for selected patients, achieving high doses in the target volume and better sparing of OAR, depending on optimal catheter placement. Because of its invasive nature, HDR-iBT requires specialized infrastructure, clinical expertise and comparatively longer treatment times, generally making SBRT easier to implement and standardize in clinical routine. Compared to SBRT_FB_, SBRT_DIBH_ achieves higher gEUD_2Gy_ and better protection of OAR due to smaller target volumes, making it a potentially better alternative when a non-invasive SBRT treatment is required. Further clinical investigations are needed to define the optimal dosing strategy for both HDR-iBT and SBRT regarding local control and clinical outcomes.

## CRediT authorship contribution statement

**Philipp Geissler:** Data curation, Visualization, Writing – original draft, Writing – review & editing. **Saskia Spautz:** Data curation, Formal analysis, Writing – original draft, Writing – review & editing. **Kathrin Hering:** Writing – original draft, Writing – review & editing. **Isabell Seiler:** Data curation, Writing – review & editing. **Frank Heinicke:** Writing – review & editing. **Ilias Sachpazidis:** Formal analysis, Writing – review & editing. **Dimos Baltas:** Conceptualization, Writing – review & editing. **Sebastian Schäfer:** Writing – review & editing, Data curation, Formal analysis. **Christos Moustakis:** Project administration, Writing – review & editing. **Nils H. Nicolay:** Conceptualization, Project administration, Writing – review & editing, Supervision. **Franziska Nägler:** Conceptualization, Data curation, Methodology, Project administration, Writing – original draft, Writing – review & editing.

## Ethics approval

This study was reviewed and approved by the Ethics Committee (432/24-ek). Informed consent to participate in the study was not required. All methods used in this study were carried out in accordance with relevant guidelines and regulations.

## Funding

This research did not receive any specific grant from funding agencies in the public, commercial, or not-for-profit sectors. Supported by the Open Access Publishing Fund of Leipzig University.

## Declaration of competing interest

The authors declare that they have no known competing financial interests or personal relationships that could have appeared to influence the work reported in this paper.

## Data Availability

The dataset generated during the current study is available from the corresponding author on reasonable request.

## References

[1] Cancer TODAY | IARC - https://gco.iarc.who.int/today. Cancer TODAY - Liver and intrahepatic bile ducts; Available from: https://gco.iarc.who.int/today.

[2] Yang A., Ju W., Yuan X., Han M., Wang X., Guo Z. (2017). Comparison between liver resection and liver transplantation on outcomes in patients with solitary hepatocellular carcinoma meeting UNOS criteria: a population-based study of the SEER database. Oncotarget.

[3] Krenzien F., Schmelzle M., Struecker B., Raschzok N., Benzing C., Jara M. (2018). Liver transplantation and liver resection for cirrhotic patients with hepatocellular carcinoma: comparison of long-term survivals. J Gastrointest Surg.

[4] Leitlinienprogramm Onkologie der Arbeitsgemeinschaft der Wissenschaftlichen Medizinischen Fachgesellschaften e. V. (AWMF), Deutschen Krebsgesellschaft e. V. (DKG) und der Stiftung Deutsche Krebshilfe. S3-Leitlinie Diagnostik und Therapie des Hepatozellulären Karzinoms und biliärer Karzinome(5.0); 2024.

[5] Akarapatima K., Chang A., Prateepchaiboon T., Pungpipattrakul N., Songjamrat A., Pakdeejit S. (2022). Comparison of overall survival between transarterial chemoembolization and best supportive care in intermediate- stage hepatocellular carcinoma. Asian Pac J Cancer Prev.

[6] Bettinger D., Pinato D.J., Schultheiss M., Sharma R., Rimassa L., Pressiani T. (2019). Stereotactic body radiation therapy as an alternative treatment for patients with hepatocellular carcinoma compared to sorafenib: a propensity score analysis. Liver Cancer.

[7] Sapir E., Tao Y., Schipper M.J., Bazzi L., Novelli P.M., Devlin P. (2018). Stereotactic body radiation therapy as an alternative to transarterial chemoembolization for hepatocellular carcinoma. Int J Radiat Oncol Biol Phys.

[8] Méndez Romero A., van der Holt B., Willemssen F.E.J.A., de Man R.A., Heijmen B.J.M., Habraken S. (2023). Transarterial chemoembolization with drug-eluting beads versus stereotactic body radiation therapy for hepatocellular carcinoma: outcomes from a multicenter, randomized, phase 2 trial (the TRENDY trial). Int J Radiat Oncol Biol Phys.

[9] Jazmati D., Boda-Heggemann J., Blanck O., Krug D. (2023). Treatment paradigms in hepatocellular carcinoma: insights gained from the TRENDY trial. Strahlenther Onkol.

[10] Alrabiah K., Liao G., Shen Q., Chiang C.-L., Dawson L.A. (2022). The evolving role of radiation therapy as treatment for liver metastases. J Natl Cancer Cent.

[11] Mahadevan A., Blanck O., Lanciano R., Peddada A., Sundararaman S., D’Ambrosio D. (2018). Stereotactic body radiotherapy (SBRT) for liver metastasis - clinical outcomes from the international multi-institutional RSSearch® Patient Registry. Radiat Oncol.

[12] Klement R.J., Guckenberger M., Alheid H., Allgäuer M., Becker G., Blanck O. (2017). Stereotactic body radiotherapy for oligo-metastatic liver disease - Influence of pre-treatment chemotherapy and histology on local tumor control. Radiother Oncol.

[13] Lee J., Shin I.-S., Yoon W.S., Koom W.S., Rim C.H. (2020). Comparisons between radiofrequency ablation and stereotactic body radiotherapy for liver malignancies: meta-analyses and a systematic review. Radiother Oncol.

[14] Collettini F., Lutter A., Schnapauff D., Hildebrandt B., Puhl G., Denecke T. (2014). Unresectable colorectal liver metastases: percutaneous ablation using CT-guided high-dose-rate brachytherapy (CT-HDBRT). Rofo.

[15] Walter F., Fuchs F., Gerum S., Rottler M.C., Erdelkamp R., Neumann J. (2021). HDR brachytherapy and SBRT as bridging therapy to liver transplantation in HCC patients: a single-center experience. Front Oncol.

[16] Auer T.A., Müller L., Schulze D., Anhamm M., Bettinger D., Steinle V. (2024). CT-guided high-dose-rate brachytherapy versus transarterial chemoembolization in patients with unresectable hepatocellular carcinoma. Radiology.

[17] Auer T.A., Anhamm M., Böning G., Fehrenbach U., Schöning W., Lurje G. (2024). Effectiveness and safety of computed tomography-guided high-dose-rate brachytherapy in treating recurrent hepatocellular carcinoma not amenable to repeated resection or radiofrequency ablation. Eur J Surg Oncol.

[18] Vogel A., Chan S.L., Dawson L.A., Kelley R.K., Llovet J.M., Meyer T. (2025). Hepatocellular carcinoma: ESMO Clinical Practice Guideline for diagnosis, treatment and follow-up. Ann Oncol.

[19] Bilski M., Korab K., Orzechowska M., Ponikowska J., Cisek P., Jereczek-Fossa B.A. (2025). Comprehensive cohort study: computer tomography-guided high-dose rate brachytherapy as metastasis-directed therapy for liver metastases from colorectal cancer in repeat oligoprogression. Radiol Med.

[20] Cisek P., Bilski M., Ponikowska J., Wojtyna E., Fijuth J., Kuncman Ł. (2025). EORTC/ESTRO defined induced oligopersistence of liver metastases from colorectal cancer - outcomes and toxicity profile of computer tomography guided high-dose-rate brachytherapy. Clin Exp Metastasis.

[21] Hass P., Mohnike K., Kropf S., Brunner T.B., Walke M., Albers D. (2019). Comparative analysis between interstitial brachytherapy and stereotactic body irradiation for local ablation in liver malignancies. Brachytherapy.

[22] Walter F., Nierer L., Rottler M., Duque A.S., Weingandt H., Well J. (2021). Comparison of liver exposure in CT-guided high-dose rate (HDR) interstitial brachytherapy versus SBRT in hepatocellular carcinoma. Radiat Oncol.

[23] Pennington J.D., Park S.J., Abgaryan N., Banerjee R., Lee P.P., Loh C. (2015). Dosimetric comparison of brachyablation and stereotactic ablative body radiotherapy in the treatment of liver metastasis. Brachytherapy.

[24] Bilski M., Korab K., Stąpór-Fudzińska M., Ponikowska J., Brzozowska A., Sroka Ł. (2024). HDR brachytherapy versus robotic-based and linac-based stereotactic ablative body radiotherapy in the treatment of liver metastases - a dosimetric comparison study of three radioablative techniques. Clin Transl Radiat Oncol.

[25] Bilski M., Peszyńska-Piorun M., Konat-Bąska K., Brzozowska A., Korab K., Wojtyna E. (2024). Radiotherapy as a metastasis directed therapy for liver oligometastases - comparative analysis between CT-guided interstitial HDR brachytherapy and two SBRT modalities performed on double-layer and single layer LINACs. Front Oncol.

[26] Dejonckheere C.S., Bilski M., Nour Y., Scafa D., Cisek P., Korab K. (2025). A dosimetric comparison of different radiotherapy modalities for non-resected oligometastatic liver disease. Clin Transl Radiat Oncol.

[27] Rivard MJ, Coursey BM, DeWerd LA, Hanson WF, Huq MS, Ibbott GS et al. Update of AAPM Task Group No. 43 Report: A revised AAPM protocol for brachytherapy dose calculations. Med Phys 2004;31:633–74. https://doi.org/10.1118/1.1646040. 10.1118/1.1646040 15070264

[28] Karagiannis E., Strouthos I., Leczynski A., Zamboglou N., Ferentinos K. (2022). Narrative review of high-dose-rate interstitial brachytherapy in primary or secondary liver tumors. Front Oncol.

[29] Goodman K.A., Kavanagh B.D. (2017). Stereotactic body radiotherapy for liver metastases. Semin Radiat Oncol.

[30] Lewis S., Barry A., Hawkins M.A. (2022). Hypofractionation in hepatocellular carcinoma - the effect of fractionation size. Clin Oncol.

[31] Timmerman R. (2022). A story of hypofractionation and the table on the wall. Int J Radiat Oncol Biol Phys.

[32] Brunner T.B., Boda-Heggemann J., Bürgy D., Corradini S., Dieckmann U., Gawish A. (2024). Dose prescription for stereotactic body radiotherapy: general and organ-specific consensus statement from the DEGRO/DGMP Working Group Stereotactic Radiotherapy and Radiosurgery. Strahlenther Onkol.

[33] Wilke L., Andratschke N., Blanck O., Brunner T.B., Combs S., Grosu A.-L. (2019). ICRU report 91 on prescribing, recording, and reporting of stereotactic treatments with small photon beams: Statement from the DEGRO/DGMP working group stereotactic radiotherapy and radiosurgery. Strahlenther Onkol.

[34] van Leeuwen C.M., Oei A.L., Crezee J., Bel A., Franken N.A.P., Stalpers L.J.A. (2018). The alfa and beta of tumours: a review of parameters of the linear-quadratic model, derived from clinical radiotherapy studies. Radiat Oncol.

[35] Fowler J.F. (2010). 21 years of biologically effective dose. Br J Radiol.

[36] Jones B., Dale R.G., Deehan C., Hopkins K.I., Morgan D.A. (2001). The role of biologically effective dose (BED) in clinical oncology. Clin Oncol.

[37] Søvik A., Ovrum J., Olsen D.R., Malinen E. (2007). On the parameter describing the generalised equivalent uniform dose (gEUD) for tumours. Phys Med.

[38] Wu Q., Mohan R., Niemierko A., Schmidt-Ullrich R. (2002). Optimization of intensity-modulated radiotherapy plans based on the equivalent uniform dose. Int J Radiat Oncol Biol Phys.

[39] Milickovic N., Tselis N., Karagiannis E., Ferentinos K., Zamboglou N. (2017). Iridium-knife: another knife in radiation oncology. Brachytherapy.

[40] Xiao Z., Zou W.J., Chen T., Yue N.J., Jabbour S.K., Parikh R. (2018). Using gEUD based plan analysis method to evaluate proton vs. photon plans for lung cancer radiation therapy. J Appl Clin Med Phys.

[41] Seidensticker M., Seidensticker R., Mohnike K., Wybranski C., Kalinski T., Luess S. (2011). Quantitative in vivo assessment of radiation injury of the liver using Gd-EOB-DTPA enhanced MRI: tolerance dose of small liver volumes. Radiat Oncol.

[42] Hansen C.R., Crijns W., Hussein M., Rossi L., Gallego P., Verbakel W. (2020). Radiotherapy treatment plannINg study guidelines (RATING): a framework for setting up and reporting on scientific treatment planning studies. Radiother Oncol.

[43] Bae S.H., Chun S.-J., Chung J.-H., Kim E., Kang J.-K., Jang W.I. (2024). Stereotactic body radiation therapy for hepatocellular carcinoma: meta-analysis and international stereotactic radiosurgery society practice guidelines. Int J Radiat Oncol Biol Phys.

[44] Franzese C., Louie A.V., Kotecha R., Zhang Z., Guckenberger M., Kim M.-S. (2025). Stereotactic body radiation therapy for liver metastases: systematic review and meta-analysis with international stereotactic radiosurgery society (ISRS) practice guidelines. Pract Radiat Oncol.

[45] Scorsetti M., Comito T., Cozzi L., Clerici E., Tozzi A., Franzese C. (2015). The challenge of inoperable hepatocellular carcinoma (HCC): results of a single-institutional experience on stereotactic body radiation therapy (SBRT). J Cancer Res Clin Oncol.

[46] Su T.-S., Liu Q.-H., Zhu X.-F., Liang P., Liang S.-X., Lai L. (2021). Optimal stereotactic body radiotherapy dosage for hepatocellular carcinoma: a multicenter study. Radiat Oncol.

[47] Cervantes A., Adam R., Roselló S., Arnold D., Normanno N., Taïeb J. (2023). Metastatic colorectal cancer: ESMO Clinical Practice Guideline for diagnosis, treatment and follow-up. Ann Oncol.

[48] Singal A.G., Llovet J.M., Yarchoan M., Mehta N., Heimbach J.K., Dawson L.A. (2023). AASLD Practice Guidance on prevention, diagnosis, and treatment of hepatocellular carcinoma. Hepatology.

[49] Stick L.B., Vogelius I.R., Risum S., Josipovic M. (2020). Intrafractional fiducial marker position variations in stereotactic liver radiotherapy during voluntary deep inspiration breath-hold. Br J Radiol.

[50] Shajid S.M., Aggarwal L.M., Mourya A., Choudhary S., Priean V.G., Singh A. (2024). A comparison of TG-43 and TG-186 dose calculation algorithms for treatment planning of intra-cavitary brachytherapy using tandem and ovoid applicator. J Contemp Brachytherapy.

[51] Boman E.L., Satherley T.W.S., Schleich N., Paterson D.B., Greig L., Louwe R.J.W. (2017). The validity of Acuros BV and TG-43 for high-dose-rate brachytherapy superficial mold treatments. Brachytherapy.

