# Supplementary material for: Comparison of interstitial high-dose-rate brachytherapy and stereotactic radiotherapy in breath-hold technique for inoperable primary and secondary liver tumors
Source: Phys Imaging Radiat Oncol. 2025 Jul 13;35:100811. doi: 10.1016/j.phro.2025.100811 (PMC12301820; doi:10.1016/j.phro.2025.100811)
Supplement: Supplementary Data 1 [file mmc1.pdf]

Supplementary Data

Supplementary table S1: OAR - dose constraints for SBRT (modified according to Timmerman et al.) and iBT treatment planning (modified according to Karaginnis et al.) [28,31]

OAR	SBRT		iBT	
	Volume	Dose limit [Gy]	Volume	Dose limit [Gy]
**Liver minus GTV**	<700.0 cm^3	17.7		
	<50.0%	15.0	66.0%	<5.0
	<30.0%	21.0	33.0%	<10.0
**Renal cortex (right + left)**	<200.0 cm^3	14.7	50.0%	<6.0
	33.0%	10.0	66.0%	<7.0
			200.0 cm^3	<10.0
			1.0 cm^3	<18.0
**Renal hilum**	<15.0 cm^3	19.5		
**Gall Bladder**			1.0 cm^3	<20.0
**Esophagus**	0.03 cm^3	32.4	1.0 cm^3	<12.0
	<5.0 cm^3	27.9	0.1 cm^3	<15.0
**Stomach**	0.03 cm^3	30.0	1.0 cm^3	<12.0
	<5.0 cm^3	22.5	0.1 cm^3	<15.0
**Duodenum**	0.03 cm^3	30.0	1.0 cm^3	<12.0
	<5.0 cm^3	22.5	0.1 cm^3	<15.0
	<10.0 cm^3	11.4		
**Small Intestine**	0.03 cm^3	28.5	1.0 cm^3	<12.0
	<30.0 cm^3	20.7	0.1 cm^3	<15.0
**Large intestine**	0.03 cm^3	45.0	1.0 cm^3	<12.0
	<20.0 cm^3	28.8	0.1 cm^3	<15.0
**Spinal Cord**	0.03 cm^3	22.5	1.0 cm^3	<10.0
	<0.35 cm^3	15.9	0.1 cm^3	<12.0
	<1.2 cm^3	12.3		
**Skin**	0.03 cm^3	33.0	1.0 cm^3	<10.0
	<10.0 cm^3	31.0		
**Great vessels**	0.03 cm^3	45.0	1.0 cm^3	<30.0
	<10.0 cm^3	39.0		
**Heart**	0.03 cm^3	30.0	1.0 cm^3	<20.0
	<15.0 cm^3	24.0		
**Whole Lung**	<5.0%	20.0		
	male: 1500.0 cm^3	10.8		
	female: 950.0 cm^3	10.8		
**Rib**	0.03 cm^3	50.0		
	<5.0 cm^3	40.0		
**Bile duct**	0.03 cm^3	36.0		

Supplementary table S2: Definition of conformity indices used for dose comparison of the 3 radioablative techniques and definition for EQD2, BED and $gEUD_{2Gy}$ [36–38,39,40]. PD=prescription dose, V=volume, PTV=planning target volume, n=number of fractions, d=dose per fraction, D=total dose

Index	Definition	Formula
**CI**	Conformity Index (CI) describes the ratio of PTV coverage to PD. VPTV PD measures the PTV volume covered by PD and VPTV describes the PTV volume (0 ≤ CN ≤ 1 – ideally the CN is 1).	$CI = \frac{V_{PTV\ PD}}{V_{\mathrm{PTV}}}$
**HTCI**	Healthy Tissue Conformity (HTCI) indicates how much of the volume outside the PTV is covered by PD. Ideally V_{PD} (volume of PD) and $V_{PTV\ PD}$ (PTV volume overlap with PD). In the best case, HTCI is 1, in the worst case, 0.	$HTCI = \frac{V_{PTV\ PD}}{V_{\mathrm{PD}}}$
**CN**	The conformation number (CN) includes both the target volume coverage and the extent of protection of normal tissue outside the PTV (0 ≤ CN ≤ 1 – ideally the CN is 1).	$CN = CI * HTCI = \frac{(V_{PTV\ PD})^2}{V_{\mathrm{PTV}} * V_{\mathrm{PD}}}$
**Dose comparison**		
**BED**	Biologically effective dose (BED) is a concept for comparing different dose fractionations and their biological effectiveness. The result is an effective dose. α/β depends on tissue, in this work, an α/β of 10 is assumed for liver tumor tissue.	$BED = \mathrm{n} * \mathrm{d}(1 + \frac{d}{\alpha/\beta})$
**EQD2**	Equivalent dose in 2 Gy (EQD2) allows each type of fractionation to be expressed in terms of the total isoeffective dose that would be required if it were delivered using conventional 2 Gy fractionation. In this work α/β of 10 is assumed for tumor tissue and 3 for the normal tissue.	$EQD2 = \mathrm{D} * (\frac{d + (\alpha/\beta)}{2 + (\alpha/\beta)})$
**$gEUD_{2Gy}$**	The generalized equivalent uniform dose ($gEUD_{2Gy}$) represents a single value that compresses the information of the DVH curve. This value helps to clarify organ biological effects of a 3D dose distribution. N is the number of dose calculation points in the observed structure. $eqd2_i^a$ indicates the fractional EQD2 dose at a point i. The parameter a describes a volume effect.	$gEUD_{2Gy} = n(\frac{1}{N}\sum_i eqd2_i^a)^{\frac{1}{a}}$

Supplementary table S3: Median dose values for target volumes of HDR-iBT, $SBRT_{FB}$ and $SBRT_{DIBH}$

			D50%	D90%	D98%	D99.9%	D2%	Dmax target	Dmax body	EQD_2	BED_{10}	$gEUD_{2Gy}$
HDR-iBT	[Gy]	Median	36.9	19.4	15.9	13.5	124.0	124.0	128.0	293.1	262.6	36.5
		Min	13.5	6.9	5.1	4.2	64.0	64.0	64.0	75.1	62.6	3.6
		Max	78.4	41.5	30.0	24.4	160.0	160.0	160.0	887.8	889.5	113.7
	[%]	Median	199.5	113.5	90.2	75.6	800.0	800.0	800.0	-	-	-
		Min	84.2	43.1	31.4	24,0	320.0	320.0	320.0	-	-	-
		Max	392.2	210.8	173.7	152.5	800.0	800.0	800.0	-	-	-
SBRT FB	[Gy]	Median	49.0	40.4	36.0	30.8	55.3	56.0	56.0	115.2	135.4	103.0
		Min	45.5	37.3	32.6	10.0	54.5	55.8	55.7	107.0	124.7	81.0
		Max	54.0	46.0	38.6	35.0	60.1	61.0	61.0	125.1	160.1	127.4
	[%]	Median	130.7	107.7	96.0	82.1	147.4	149.2	149.2	-	-	-
		Min	121.4	99.5	87.0	26.7	145.4	148.8	148.5	-	-	-
		Max	144.1	122.6	102.9	93.4	160.2	162.6	162.6	-	-	-
SBRT DIBH	[Gy]	Median	49.6	40.4	35.9	30.4	55.4	56.0	56.0	116.2	138.4	107.5
		Min	46.8	36.2	17.7	7.3	54.4	55.8	55.8	107.0	127.9	66.5
		Max	53.0	50.1	47.4	43.7	58.5	59.5	59.5	127.2	159.9	128.2
	[%]	Median	132.3	107.8	95.8	81.0	147.7	149.2	149.2	-	-	-
		Min	124.9	96.6	47.1	19.5	145.0	148.9	148.9	-	-	-
		Max	141.3	133.6	126.5	116.4	155.9	158.8	158.8	-	-	-

Supplementary table S4: Median dose values for OAR: HDR-iBT, $SBRT_{FB}$ and $SBRT_{DIBH}$

			Liver V5Gy [cm³]	Liver V10Gy [cm³]	Liver V16.2Gy [cm³]	Liver Dmean [Gy]	liver D66% [Gy]	Eso. Dmean [Gy]	Stomach Dmean [Gy]	Duodenum Dmean [Gy]	Kidney_R Dmean [Gy]
HDR-iBT	absolute	Med	421.4	181.0	97.1	5.1	1.5	1.1	0.4	1.0	0.8
		Min	76.2	31.8	16.1	1.3	0.3	0.1	0.1	0.2	0.1
		Max	1662.5	1141.7	704.3	10.6	10.8	3.9	2.3	6.2	4.1
	absolute EQD2	Med	-	-	-	8.2	1.4	0.9	0.3	0.8	0.6
		Min	-	-	-	1.1	0.2	0.1	0.1	0.1	0.1
		Max	-	-	-	28.8	29.7	5.4	2.4	11.0	5.9
	Relative (%)	Med	-	-	-	27.1	-	6.1	2.1	5.5	4.8
		Min	-	-	-	8.5	-	0.9	0.6	1.4	0.7
		Max	-	-	-	63.2	-	19.5	11.5	30.4	25.8
SBRT FB	Absolute	Med	867.6	664.8	453.1	11.1	2.3	3.3	2.1	0.7	0.8
		Min	403.4	196.9	93.5	4.8	0.4	0.1	0.3	0.0	0.0
		Max	2084.8	1345.6	953.2	24.1	17.5	12.0	9.1	17.6	10.9
	absolute EQD2	Med	-	-	-	14.8	1.7	2.7	1.5	0.5	0.5
		Min	-	-	-	4.5	0.2	0.1	0.2	0.0	0.0
		Max	-	-	-	53.3	30.8	16.7	11.0	31.1	14.5
	Relative (%)	Med	-	-	-	29.5	-	8.9	5.5	1.9	2.2
		Min	-	-	-	12.9	-	0.2	0.7	0.0	0.0
		Max	-	-	-	64.4	-	31.9	24.2	46.8	29.1
SBRT DIBH	Absolute	Med	685.9	503.4	333.8	8.3	1.0	2.3	1.5	0.4	0.4
		Min	231.4	96.4	41.4	2.4	0.2	0.0	0.1	0.0	0.0
		Max	1610.2	1030.9	760.6	19.6	13.5	9.7	6.6	13.0	10,0
	absolute EQD2	Med	-	-	-	9.5	0.7	1.8	1.1	0.2	0.3
		Min	-	-	-	1.8	0.1	0.0	0.1	0.0	0.0
		Max	-	-	-	37.2	20.2	12.1	6.9	19.0	12.7
	Relative (%)	Med	-	-	-	22.0	-	6.2	4.1	1.0	1.1
		Min	-	-	-	6.4	-	0.1	0.4	0.0	0.0
		Max	-	-	-	52.1	-	25.9	17.7	34.6	26.6

Supplementary table S4 (continuation): Median dose values for OAR: HDR-iBT, $SBRT_{FB}$ and $SBRT_{DIBH}$

			Eso. D1cm³ [Gy]	Stomach D1cm³ [Gy]	Duod. D1cm³ [Gy]	Small Intest. D1cm³ [Gy]	Large Intest. D1cm³ [Gy]	Gall Bladder D1cm³ [Gy]	Spinal Cord D1cm³ [Gy]	Heart D1cm³ [Gy]	Skin D1cm³ [Gy]
**HDR-iBT**	absolute	Med	1.5	0.9	2.1	0.5	1.4	3.9	0.8	2.8	2.8
		Min	0.0	0.0	0.0	0.0	0.0	0.0	0.0	0.0	0.0
		Max	5.9	10.7	13.6	15.4	13.1	20.4	5.5	22.1	9.0
	absolute EQD2	Med	1.3	0.7	2.1	0.3	1.3	5.3	0.6	3.3	3.2
		Min	0.0	0.0	0.0	0.0	0.0	0.0	0.0	0.0	0.0
		Max	10.4	29.4	45.2	56.8	42.0	95.1	9.4	110.9	21.4
**SBRT FB**	Absolute	Med	6.3	5.1	3.0	1.1	7.3	10.7	3.5	6.0	14.8
		Min	0.2	1.7	0.0	0.0	0.0	0.4	1.5	0.1	7.7
		Max	20.7	34.0	49.1	45.1	37.6	51.4	10.1	54.3	38.1
	absolute EQD2	Med	6.5	4.8	2.4	0.8	8.0	14.0	2.9	5.9	23.4
		Min	0.1	1.2	0.0	0.0	0.0	0.2	1.0	0.1	8.6
		Max	41.0	97.6	190.3	162.9	116.8	206.8	12.9	228.8	119.8
**SBRT DIBH**	Absolute	Med	5.2	4.1	1.6	0.5	3.7	7.3	3.1	3.5	13.2
		Min	0.1	1.4	0.0	0.0	0.0	0.0	1.2	0.0	5.6
		Max	16.1	25.9	43.9	33.3	28.1	47.0	8.8	51.9	23.2
	absolute EQD2	Med	5,0	3.6	1.1	0.3	3.1	8.0	2.5	2.9	19.4
		Min	0.1	0.9	0.0	0.0	0.0	0.0	0.8	0.0	5.4
		Max	27.0	60.4	154.6	93.8	69.7	175.5	10.4	210.8	49.8

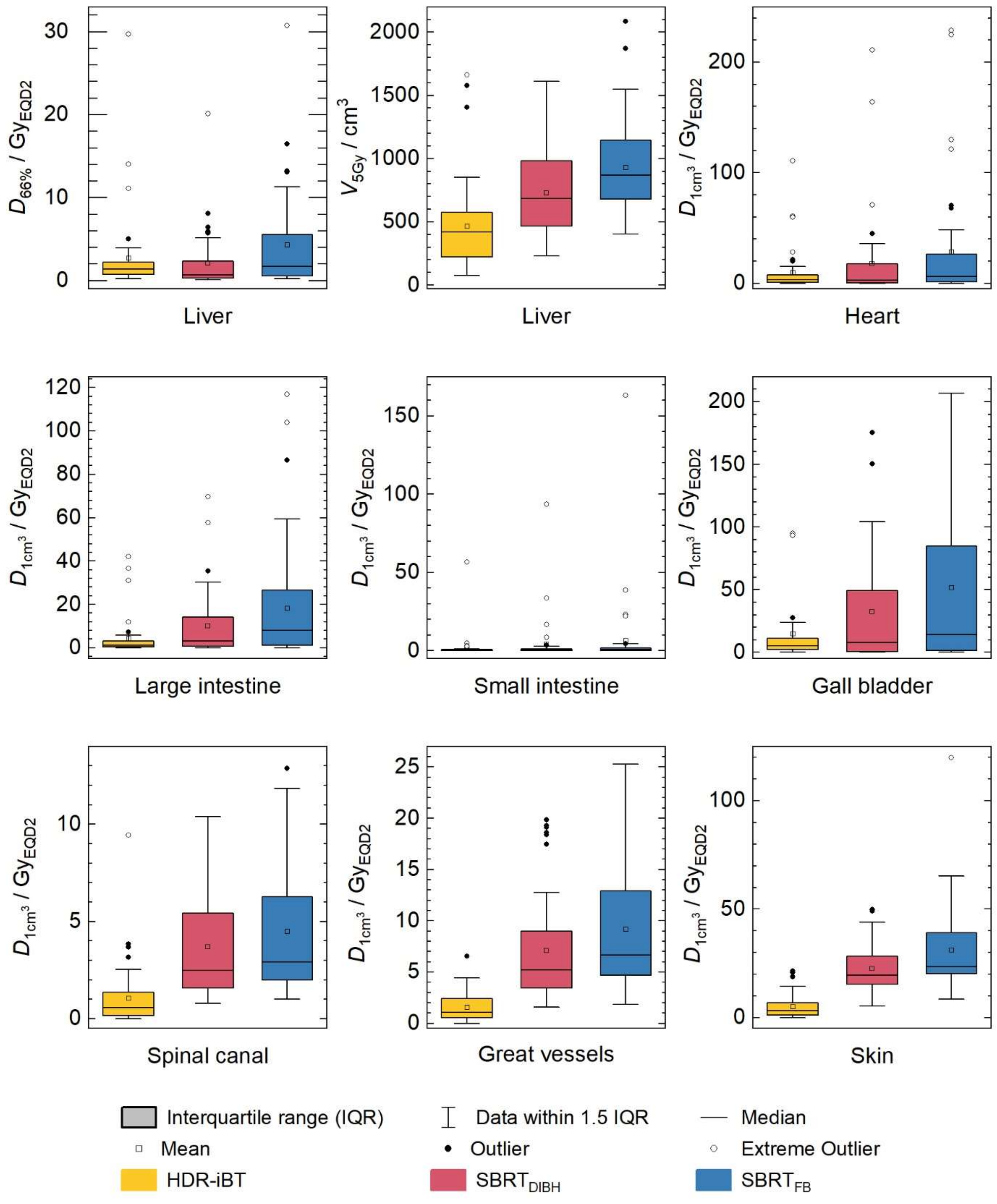

Supplementary figure S1: Dose distribution to selected organs at risk
