# Supplementary figures and images for: Comparison of interstitial high-dose-rate brachytherapy and stereotactic radiotherapy in breath-hold technique for inoperable primary and secondary liver tumors
Source: Phys Imaging Radiat Oncol. 2025 Jul 13;35:100811. doi: 10.1016/j.phro.2025.100811 (PMC12301820; doi:10.1016/j.phro.2025.100811)

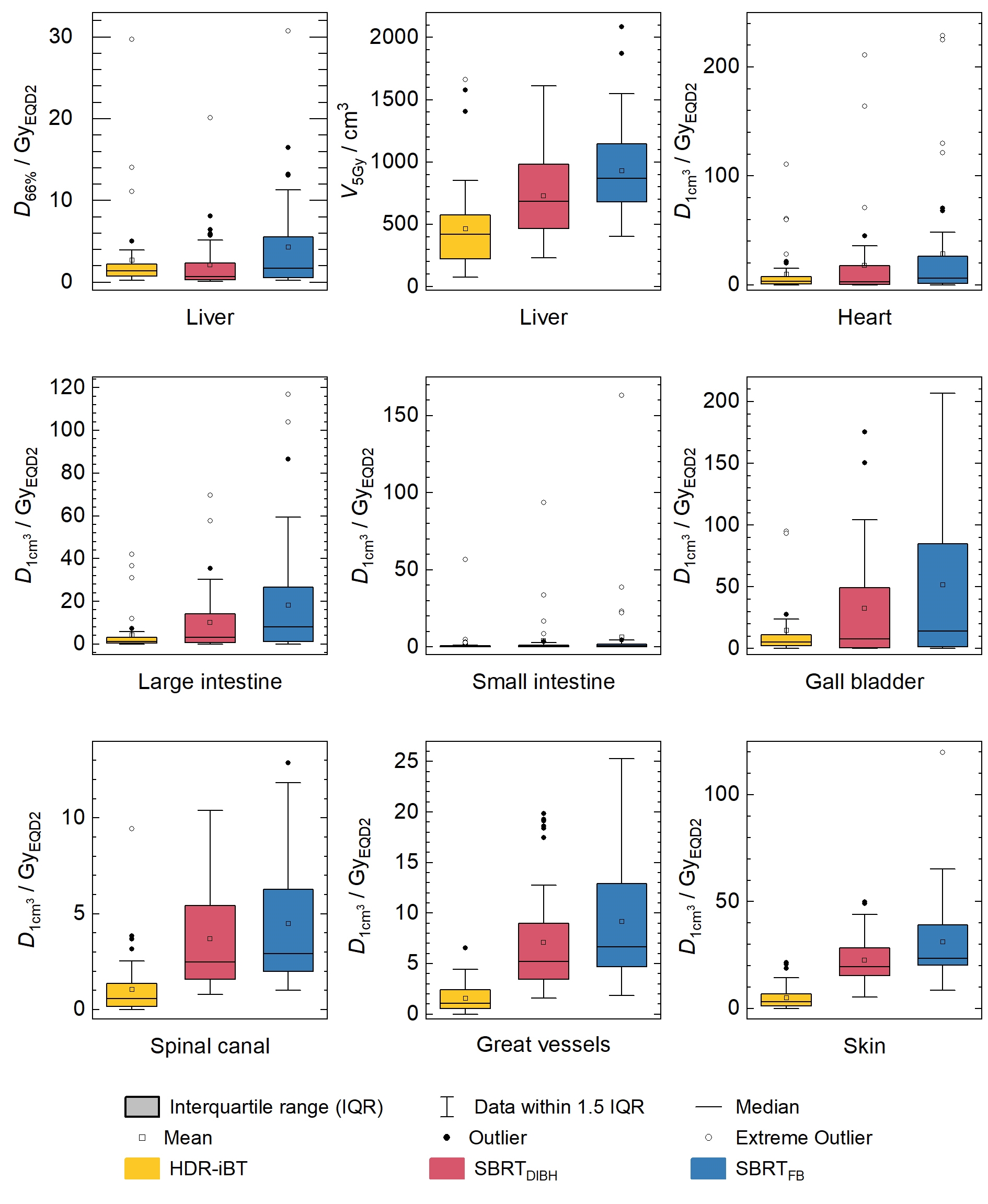

Supplement: Supplementary Figure 2 [file mmc2.jpg]
